# Ocular Morbidity among Patients Visiting the Department of Ophthalmology during the Coronavirus Disease 2021 Pandemic at a Tertiary Care Hospital: A Descriptive Cross-sectional Study

**DOI:** 10.31729/jnma.7107

**Published:** 2021-12-31

**Authors:** Pradeep Bastola, Polina Dahal

**Affiliations:** 1 Department of Ophthalmology, Chitwan Medical College, Bharatpur-10, Nepal

**Keywords:** *covid-19*, *dry eye*, *non-communicable diseases*, *refractive errors*, *visual acuity*

## Abstract

**Introduction:**

Due to the ongoing coronavirus disease 2021 pandemic and lockdown, eye care services have been compromised globally. The magnitude of ocular diseases across all populations in Nepal are few and far between and rare during this pandemic. This study was aimed to find out the prevalence of ocular morbidity among patients visiting the department of Ophthalmology of a tertiary care hospital during the pandemic.

**Methods:**

A descriptive cross-sectional study was conducted among the patients visiting the department of Ophthalmology of a tertiary care hospital from 18 August 2021 to 30 September 2021. Ethical clearance was taken from the Institutional Review Committee (Reference: 078/079023). Convenience sampling was done. Basic demographic data, clinical characteristics, visual status and prevalence of ocular morbidities were noted. Data entry was done using Statistical Package for the Social Sciences version 26. Point estimate at 95% Confidence Interval was calculated along with frequency and percentage for binary data.

**Results:**

Out of 650 study subjects examined, 454 (69.8%) (66-73.0 at 95% Confidence Interval) study subjects had at least one ocular morbidity in at least one eye. Refractive error 153 (33.7%) was the commonest ocular morbidity followed by headache 52 (11.5%), dry eyes 50 (11%), non-communicable diseases related ocular morbidity 41 (9%), and age related cataract 37 (8.1%).

**Conclusions:**

The prevalence of ocular morbidity in our study was higher than findings from other similar studies done at national and international levels, though the causes of ocular morbidity was similar.

## INTRODUCTION

According to the World Health Organization (WHO), World Report on Vision, globally 2.2 billion people have visual impairment or blindness, and 1.2 billion of them could have been addressed or prevented with proper interventions and programs in place.^1^

The coronavirus disease 2019 (COVID-19) has exposed the health systems of many countries including Western and Lower-middle-income countries like Nepal, the effect of COVID-19 and lockdown has been severe.^2^ The hospital visits by the patients for ocular problems have changed dramatically due to the COVID-19 effect. Studies depicting the magnitude of ocular diseases across all populations in Nepal are few and far between and rare during COVID-19 era.^3–6^

This study was aimed to find the prevalence of ocular morbidity among patients visiting the Ophthalmology outpatient department (OPD) of Chitwan Medical College (CMC) - Teaching Hospital during the second wave of COVID-19 pandemic and lockdown restrictions.

## METHODS

This descriptive cross-sectional study was done in Chitwan Medical College (CMC) - Teaching Hospital among the patients visiting the Department of Ophthalmology. After obtaining ethical clearance from Institutional Review Committee (IRC), (Ref: CMC-1RC/078/079-023) the study was commenced and conducted over a period of one month from 18 August - 30 September 2021 post IRC approval during COVID-19 pandemic with lockdown measures on. All patients visiting the department of Ophthalmology willing to get enrolled in the study were included in the study. The patients who were not willing, who did not provide an informed consent, patients with chronic debilitating systemic conditions, patients encountered in emergency wards were excluded from the study. Convenience sampling technique was used, and the sample size was calculated using the formula,

n = Z^2^ × p × q / e^2^

  = (1.96)^2^ × 0.155 × (1-0.155) / (0.04)^2^

  = 306

Where,

n= required sample size,
Z= 1.96 at 95% Confidence Interval (CI)
p= prevalence of ocular morbidity reported by a similar study, 15.5%^6^
q = 1-p
e = margin of error, 4%

The required sample size was 306. As convenience sampling was done, the sample size was doubled to 612. Considering non-respondent rate of 5%, total sample size becomes 643. However, our study included 650 study subjects.

In a safe environment with strict COVID-19 precautions protocol set by CMC-Teaching Hospital, the study subjects were examined. When in doubt, and before admission the study subjects were advised to undergo a real-time polymerase chain reaction (RT-PCR) test for COVID-19 infection. After explaining the purpose of the study and confidentiality of data collection, informed consent was obtained from each participant or parent/guardian in the case of a small child. The study participants were evaluated in detail in the following sequence: visual acuity measurement of each eye separately (unaided and with a pinhole), extra-ocular movement assessment, cover test, cover-uncover test, refraction using a Heine Beta 200 retinoscope, anterior segment examination with a slit lamp, and dilated fundus examination using an indirect ophthalmoscope using +90 D Volk lens, Tropicamide eye drops were used for fundus dilatation. Keith Wagener and Barker's classification of Hypertensive Retinopathy (HR)^7^ was used to grade the HR status.

In addition, a new proposed classification for HR based on fundus findings correlated with optical coherence tomography findings where applicable was also used to detect accurate retinopathy status.^8^

The stages of diabetic retinopathy were graded using Early Treatment Diabetic Retinopathy Study^9^ (ETDRS). Schirmer's test, tear film break-up time (TBUT), clinical activity score for thyroid eye disease patients, fluorescein staining, intraocular pressure (IOP) measurement, syringing were done in subjects where indicated.

The participants were advised to undergo investigations and or admissions when and where necessary. The patients not willing to provide informed consent were automatically excluded from the study. A refractive error diagnosis was made in the following way, for myopia any refractive error more than -0.5Dioptre (D) and for hypermetropia +1.0D. An interobserver value of Kappa was used for the validity of diagnosis and any value more than 0.8 was considered strong agreement.

Statistical Package for the Social Sciences (SPSS) version 26 was used for data entry and analysis. Descriptive statistics were applied, and results were expressed as frequencies whereas continuous variables were expressed as mean±SD or median. Point estimate at 95% CI was calculated along with frequency and percentage for binary data.

## RESULTS

Out of 650 study subjects were examined, 454 (69.8%) (66-73.0 at 95% Confidence Interval) study subjects had at least one ocular morbidity in at least one eye. Refractive error 153 (33.7%) was the commonest ocular morbidity followed by headache due to Ophthalmic cause 52 (11.5%), dry eyes 50 (11%) non-communicable diseases related ocular morbidity 41 (9%) and age-related cataract 37 (8.1%) (Table 1).

**Table 1:** Showing the prevalence and causes of ocular morbidity during COVID-19 pandemic and lockdown restrictions in study subjects.

Variables	n (%)
Refractive error	153 (33.7)
Dry eyes	50 (11.0)
Ocular allergy	12 (2.6)
Age related cataract	37 (8.1)
Glaucoma	5 (1.1)
Diabetic retinopathy	11 (2.4)
Simple/viral conjunctivitis	10 (2.2)
Corneal ulcer	2 (0.4)
Hypertensive retinopathy	15 (3.3)
Third nerve palsy	4 (0.9)
Orbital cellulitis	1 (0.2)
Computer vision syndrome	26 (5.7)
Presbyopia	16 (3.5)
Intraorbital abscess	1 (0.2)
Sclerouveitis	4 (0.9)
Uveitis	3 (0.7)
Acute dacrocystitis	2 (0.4)
Milia	1 (0.2)
Pterygium	9 (2.0)
Thyroid Eye Disease	15 (3.3)
Chalazion	7 (1.5)
Herpes Zoster Ophthalmicus	8 (1.8)
Common Migraine/Classical Migraine	52 (11.5)
Idiopathic intracranial hypertension (Pseudotumor cerebri)^*^	3 (0.7)
Episcleritis	2 (0.4)
Bell's Palsy	1 (0.2)
Preseptal cellulitis/Lid abscess	1 (0.2)
Age related macular degeneration	1 (0.2)
Intra-orbital tumor	1 (0.2)
Idiopathic orbital inflammation (Pseudotumor oculi)	1 (0.2)
**Total**	454 (100)

* All three cases of pseudotumor cerebri had COVID-19 infection.

The mean age of presentation was 45.1 years (1.90±17.3). Gender wise females 290 (63.9%) outnumbered the males. Most of the study participants were from urban areas 394 (86.8%). Seventy-two (15.8%) study subjects had a history of COVID-19 infection or were currently positive whereas, 29 study subjects (6.38%) were not aware about their COVID-19 infection status. Occupation wise housewives 190 (41.9%), farmers 74 (16.3%), and students 43 (9.5%) were the common study subjects. Two-hundred and twenty-one (48.7%) were vaccinated with at least a dose of COVID-19 vaccine (Table 2).

**Table 2:** Showing the demographic characteristics of the study subjects with ocular morbidities (n = 454).

Gender distribution	n (%)
Male	164 (36.1)
Female	290 (63.9)
Total	454 (100)
**Address of the study subjects**	
Urban	394 (86.8)
Rural	60 (13.2)
Total	454 (100)
**Occupation of the study subjects**	
Abroad	4 (0.9)
Army	3 (0.7)
Banker	5 (1.1)
Businessman	29 (6.4)
Carpenter	1 (0.2)
Child	4 (0.9)
Driver	6 (1.3)
Farmer	74 (16.3)
Housewife	190 (41.9)
Labour	1 (0.2)
NGO	4 (0.9)
Nurse	7 (1.5)
Office work	6 (1.3)
Pharmacist	3 (0.7)
Priest	1 (0.2)
Retired	29 (6.4)
Shopkeeper	19 (4.2)
Student	43 (9.5)
Tailoring	3 (0.7)
Teacher	14 (3.1)
Unemployed	6 (1.3)
Waiter	2 (0.4)
Total	454 (100)
**Past or current covid-19 infection status**	
Yes	72 (15.9)
No	353 (77.7)
Do not know	29 (6.4)
Total	454 (100)
**Covid-19 vaccination status**	
Vaccinated (at least one dose)	221 (48.7)
Not vaccinated	233 (51.3)
**Total**	454 (100)

When the clinical characteristics of the study subjects were analysed, 385 (84.8%) of the study subjects had normal visual acuity (best corrected), 368 (81.1 %) study subjects had normal anterior segment, 449 (98.9%) of the study subjects had normal pupillary reaction and 413 (91.0%) of the study subjects presented with normal fundus (Table 3).

**Table 3:** Showing the clinical characteristics and ocular findings in the study subjects with ocular morbidities (n = 454).

Visual acuity with best possible correction	n (%)
Normal	385 (84.8)
Mild VI	34 (7.5)
Moderate VI	19 (4.2)
Severe VI	9 (2.0)
Blind	7 (.7)
Total	454 (100)
**Aneterior segment examination findings**	
Normal	368 (81.1)
Anterior segment pathology	86 (18.9)
Total	454 (100)
**Pupillary reactions**	
Normal size, normal reaction	449 (98.9)
RAPD	4 (.9)
Afferent pupillary defect (APD)	1 (.2)
Total	454 (100)
**Fundus examination findings**	
Normal	413 (91.0)
Hypertensive retinopathy	15 (3.3)
Diabetic retinopathy	11 (2.4)
Papilledema	4 (.9)
Glaucomatous optic disc	5 (1.1)
Other fundus pathology	6 (1.3)
**Total**	454 (100)

Fifteen (3.3%) study subjects and 11 (2.4%) study subjects had hypertensive and diabetic retinopathy (Table 4).

**Table 4:** Showing the grading of hypertensive and diabetic retinopathy in the study subjects (n=454).

Grade of hypertensive retinopathy	n (%)
Grade 0	439 (96.7)
Grade 1	9 (1.9)
Grade 2	3 (0.7)
Grade 3	3 (0.7)
Total	454 (100)
**Findings of Diabetic retinopathy (n = 454)**	**n (%)**
Normal	443 (97.7)
Mild non proliferative diabetic retinopathy (NPDR)	4 (0.9)
Moderate NPDR	1 (0.2)
Severe NPDR	1 (0.2)
Very Severe NPDR	1 (0.2)
Proliferative Diabetic retinopathy	1 (0.2)
Diabetic eye disease (DED)	2 (0.4)
Clinically significant macular edema	1(0.2)
**Total**	**454 (100)**

## DISCUSSION

A total of 650 subjects were enrolled in the study, 1300 eyes were evaluated in detail. The mean age of presentation was 45.1 years (1-90±17.3). Gender wise females outnumbered the males. The demographic findings of the current study (Table 2) were comparable to a study done by Veronika Y, et al. which concluded that; the demographics of the patients visiting Ophthalmic emergency room was same in years 2019 (pre COVID era) and 2020 (COVID-19 era), however the number of patients visiting the department was declining.^10^

The prevalence of ocular morbidity in the current was found to be high 454 (69.5%), this finding from the study did not relate with a study done elsewhere^6^ where the prevalence of ocular morbidity was found to be 15.5%, this could have been attributed by the fact that this study was a hospital based study done during peak COVID-19 pandemic, so only patients needing Ophthalmic care must have visited the hospital during the study period (Table 1).

When looking at the causes of ocular morbidity and prevalent ocular diseases, the study findings hinted towards a gentle rise in non-communicable diseases associated ocular morbidity, and emerging rarer ocular morbidities like pseudotumor cerebri, psuedotumor oculi, orbital cellulitis, intraorbital abscess, and intraorbital tumour (Table 1). These findings in the study were comparable with various other studies done elsewhere.^11-15^ The findings from the current study also suggested that refractive error (33.7%) and dry eyes (11.7%) are still the leading causes of ocular morbidity in both COVID-19 infected and non-infected study subjects. High prevalence of refractive error, dry eyes and to some extent computer vision syndrome (Table 1) in the study was attributed by the number of hours general population needed to stay at home and work through digital and social media during the pandemic and lockdown, this finding of the study also related well with pre COVID-19 era study done by Bastola P where refractive error and dry eyes were the most prevalent ocular diseases.^16^ The lower number of age-related cataracts (8.1%) in the study subjects might be due to the slow progression of disease and lack of hospital visits due to accessibility issue in lockdown specially for the senile (elderly) population.

Clinical characteristics of the study subjects like visual acuity, anterior segment findings and posterior segment findings in the present study (Table 3) were indicative of the trends in ocular morbidity and these features were comparable with a study done in pre COVID era.^16^ The evolution of positive clinical findings due to non-communicable diseases like hypertension, diabetes mellitus, thyroid eye disorders, classical and common migraine (Table 1, 3) in the study has been explained well by comparable studies done elsewhere.^11-13^ These findings were supported by the fact that due to COVID-19 pandemic and lockdown measures; the life style of general population was altered and modified which led to a more sedentary life style leading to aggravation of pre-existing hypertension, diabetes and thyroid disorder and new onset diabetic retinopathy and hypertensive retinopathy with mental health issues. In addition, the patients with diabetes and hypertension missed their routine follow ups for Ophthalmic examination during the COVID-19 pandemic and lockdown.^13^

Not a single COVID-19 positive study subject in our study revealed retinal findings suggestive of COVID-19 retinopathy. This finding of our study differed from a study done by Bansal R, et al. who reported cases of retinal haemorrhage non-specific to diabetic or hypertensive retinopathy and proposed a terminology as COVID-19 retinopathy.^14^ This could be attributed to a less severe form of COVID-19 infection in the COVID-19 positive study subjects due to vaccination status.

The reported prevalence of ocular manifestations of COVID-19 infection from various studies is 2-32%, in our study the ocular manifestations and morbidity due to COVID-19 infected subjects was 15.8%, this finding from our study was comparable with existing literature.^17-22^ In the current study, a relatively rare condition like pseudotumor cerebri was seen in three of the study participants. This was comparable to the findings from a study done by Noro F, et al. who explained and reported pseudotumor cerebri post COVID-19 infection as a persistent cause of headache.^23^ The rarer ocular manifestations and morbidity of COVID-19 infection in the present study needed admission and further management. These findings in our study would need to get explored more to find out whether these rarer conditions were COVID-19 related or were isolated diagnosis.

Majority of the study subjects within this study belonged to urban areas, this finding in the study can be explained by the lockdown measures and COVID-19 restrictions, which meant patients living nearby to the study hospital had access to visit the hospital. However, patients from rural areas could not visit the hospital as mobility was strictly prohibited. Most of the study subjects being females, farmers and students was attributed by the workload they had to bear during the restrictions, strict lockdown and the physical and mental stress they had to go through during COVID-19 pandemic. The youths and students forming larger number of study subjects in the current study was attributed by online studies, webinars and use of various social media and digital platforms during COVID-19 era leading to eye related problems and digital eye strain which related well with the findings from a study done elsewhere.^11^ At least a single dose vaccination status in 221 (48.7%) study subjects showed the robust vaccination campaign Nepal adopted and their success during the hard times of second wave of COVID-19 pandemic (Table 2).

The study was done in a tertiary hospital of urban, central Nepal thus the study sample may not be representable to other parts of Nepal. We would like to recommend further, to explore and find out the changing ocular morbidity and prevalence of ocular diseases during COVID-19 era by doing similar studies in other parts of Nepal.

## CONCLUSIONS

The prevalence of ocular morbidities in our study was higher than findings from similar study done at national and international study, though this study was done during COVID-19 pandemic with strict lockdown measures at the hospital. The study could also conclude that refractive error remains the most prevalent ocular morbidity and visual impairment followed by migraine headache, dry eyes and ocular morbidity due to systemic non-communicable diseases. Non-communicable diseases related ocular morbidities have been more prevalent during COVID-19 pandemic. Relatively rarer ocular conditions like pseudotumor oculi, intraorbital abscess, pseudotumor cerebri, cranial nerve palsies, and orbital cellulitis have been diagnosed to be ocular manifestations of COVID-19 infection, and these conditions should be managed judiciously and may need admission. The study could also conclude dry eyes and computer vision syndromes to be more prevalent during these times.
